# Global variation in skin injures and skincare practices in extremely preterm infants

**DOI:** 10.1007/s12519-022-00625-2

**Published:** 2022-11-13

**Authors:** Pranav Jani, Umesh Mishra, Julia Buchmayer, Rajesh Maheshwari, Daphne D’Çruz, Karen Walker, Duygu Gözen, Krista Lowe, Audrey Wright, James Marceau, Mihaela Culcer, Archana Priyadarshi, Adrienne Kirby, James E. Moore, Ju Lee Oei, Vibhuti Shah, Umesh Vaidya, Abdelmoneim Khashana, Sunit Godambe, Fook Choe Cheah, Wen-Hao Zhou, Xiao-Jing Hu, Muneerah Satardien

**Affiliations:** 1 Faculty of Medicine and Health, The University of Sydney, Sydney, NSW Australia; 2 Department of Neonatology, Westmead Hospital, Westmead, NSW 2145 Australia; 3 Comprehensive Center for Pediatrics, Department of Pediatrics and Adolescent Medicine, Division of Neonatology, Pediatric Intensive Care and Neuropediatrics, Medical University of Vienna, Vienna, Austria; 4 Department of Newborn Care, Royal Prince Alfred Hospital, Camperdown, NSW Australia; 5 Council of International Neonatal Nurses, Boston, MA USA; 6 The George Institute for Global Health, Sydney, NSW Australia; 7 Sydney Institute for Women, Children and Their Families, Sydney, NSW Australia; 8 Pediatric Nursing Department, Florence Nightingale Faculty of Nursing, İstanbul University-Cerrahpaşa, Istanbul, Turkey; 9 The National Health and Medical Research Council Clinical Trials Centre, University of Sydney, Sydney, NSW Australia; 10 Connecticut Children’s Division of Neonatal-Perinatal Medicine, Connecticut Children’s Medical Center, Hartford, CT USA; 11 UCONN School of Medicine, Farmington, CT USA; 12 The Royal Hospital for Women, Randwick, NSW Australia; 13 School of Women’s and Children’s Health, University of New South Wales, Randwick, NSW Australia; 14 Department of Paediatrics and Institute of Health Policy, Management and Evaluation, Mount Sinai Hospital, Toronto, Canada; 15 Department of Pediatrics, King Edward Memorial Hospital, Pune, India; 16 Pediatrics, Suez Canal University, Ismailia, Egypt; 17 Divisional Director for Clinical Governance, Women’s, Children’s and Clinical Support, Imperial College Healthcare NHS Trust, London, UK; 18 Department of Pediatrics, Faculty of Medicine, Universiti Kebangsaan, Bangi, Malaysia; 19 Hospital Canselor Tuanku Muhriz, Jalan Yaacob Latif, Bandar Tun Razak, Kuala Lumpur, Malaysia; 20 Department of Neonatology and Vice President, Children’s Hospital of Fudan University, Shanghai, China; 21 Vice Director of Nursing Department, Children’s Hospital of Fudan University, Shanghai, China; 22 Department of Paediatrics and Child Health, Tygerberg Hospital, Cape Town, South Africa; 23 University of Stellenbosch, Cape Town, South Africa

**Keywords:** Extremely premature infants, Injuries, Neonatal intensive care unit, Skin care, Wounds

## Abstract

**Background:**

Globally, are skincare practices and skin injuries in extremely preterm infants comparable? This study describes skin injuries, variation in skincare practices and investigates any association between them.

**Methods:**

A web-based survey was conducted between February 2019 and August 2021. Quantifying skin injuries and describing skincare practices in extremely preterm infants were the main outcomes. The association between skin injuries and skincare practices was established using binary multivariable logistic regression adjusted for regions.

**Results:**

Responses from 848 neonatal intensive care units, representing all geographic regions and income status groups were received. Diaper dermatitis (331/840, 39%) and medical adhesive-related skin injuries (319/838, 38%) were the most common injuries. Following a local skincare guideline reduced skin injuries [medical adhesive-related injuries: adjusted odds ratios (aOR) = 0.63, 95% confidence interval (CI) = 0.45–0.88; perineal injuries: aOR = 0.66, 95% CI = 0.45–0.96; local skin infections: OR = 0.41, 95% CI = 0.26–0.65; chemical burns: OR = 0.46, 95% CI = 0.26–0.83; thermal burns: OR = 0.51, 95% CI = 0.27–0.96]. Performing skin assessments at least every four hours reduced skin injuries (abrasion: aOR = 0.48, 95% CI = 0.33–0.67; pressure: aOR = 0.51, 95% CI = 0.34–0.78; diaper dermatitis: aOR = 0.71, 95% CI = 0.51–0.99; perineal: aOR = 0.52, 95% CI = 0.36–0.75). Regional and resource settings-based variations in skin injuries and skincare practices were observed.

**Conclusions:**

Skin injuries were common in extremely preterm infants. Consistency in practice and improved surveillance appears to reduce the occurrence of these injuries. Better evidence regarding optimal practices is needed to reduce skin injuries and minimize practice variations.

## Introduction

The skin plays a vital role in the protection, thermoregulation, and sensory functions of the body [1]. Extremely preterm (EP) infants, born < 28 weeks gestational age (GA), are vulnerable to thermal imbalance, fluid and electrolyte loss, skin injury, and sepsis arising from wound contamination and skin breakdown, all due to developmental immaturity of the skin [2, 3]. Hence, it is imperative that the best evidence-based practices are implemented to promote skin integrity and reduce complications. EP infants may also develop injuries from mechanical causes, and from intensive care practices [4]. The prevalence of skin injuries in term and preterm infants ranges from 9.25% to 43.1% [5].

The influence of geographic region and resource settings on skincare practices, and whether skincare practices for EP infants are comparable across the globe in the delivery of evidence-based medicine are unknown. This international survey was designed to provide a comprehensive description of skin injuries, identify variation in skincare practices, and ascertain any association between these practices and skin injuries in EP infants. We hypothesized that significant variation in practice exists, and that skin injuries occurred frequently with certain practices. The findings of this study have implications for advancing the understanding of skincare practices and therefore improving healthcare delivery and clinical outcomes.

## Methods

Neonatal intensive care units (NICUs) providing care to EP infants were identified, either directly from an internet search or through regional professional neonatal or parent organizations. The NICU directors were then contacted by an email and invited to participate.

Research Electronic Data Capture (REDCap, Vanderbilt University, Nashville, TN, USA) was used to create a secure e-questionnaire and capture the responses. The link to access the questionnaire was included in the request-to-participate letter. Most questions were closed ones (either single or multiple-choice), few were open to allow for free text descriptions of other practices or commercial products. This international survey was an extension of a pilot study conducted in Australia and New Zealand [6]. Ethical approval was obtained before commencing the study (LNR/18/WMEAD/288–5770 and REB-20-0213-E). Information regarding the purpose of the study, names of the investigators, informed consenting process, time for completion of the survey, security of data storage, and protection of participants’ privacy was provided in the request-to-participate invitation letter. Participation in the survey was voluntary, and participants consented by clicking “Yes–I agree to participate”. Only one response per NICU was requested. To increase participation in the survey, a reminder was sent twice after the initial invitation. A 5-point unipolar scale was used to record the occurrence of skin injuries. This information was then dichotomized to uncommon (including the responses rare or seldom) and common (often, almost always and always).

### Statistics

Data were analyzed using Stata 17 (StataCorp, College Station, TX, USA). Descriptive statistics were used to summarize the responses. Chi-squared test or where appropriate Fisher’s exact test was used to explore region and income status-based differences in skincare practices. The association between skin injuries and skincare practices was first assessed in univariate models. Binary multivariable logistic models adjusted for regions including variables that had a *P* value of < 0.2 in the univariate models were then created using backward stepwise selection. Results from these models are reported with adjusted odds ratios (aOR) and 95% confidence intervals (CI) (Table 1). A two-tailed *P* value < 0.05 was considered as significant and no adjustments have been made for multiple comparisons.

**Table 1:** Relationship between skin care practices and skin injuries from binary univariate and multivariable logistic regression

Practices or variables	Yes/no	Occurrence of injuries		Univariate, OR (95% CI)	*P*	Multivariable, aOR (95% CI)	*P*
		Uncommon (rare/seldom), *n* (%)	Common (often/almost always/always), *n* (%)				
MARSI							
Local skin care guideline available	No	117 (53)	104 (47)	0.58 (0.42–0.80)	0.001	0.63 (0.45–0.88)	0.008
	Yes	378 (66)	197 (34)				
Tapes used for securing tubes to the skin							
Transparent film dressing	No	323 (62)	199 (38)	0.99 (0.74–1.32)	0.960	NA	NA
	Yes	196 (62)	120 (38)				
Hydrocolloid base with transparent adhesive tape	No	340 (61)	219 (39)	0.86 (0.64–1.16)	0.340	NA	NA
	Yes	179 (64)	100 (36)				
Silicone tapes	No	429 (61)	271 (39)	0.84 (0.57–1.23)	0.380	NA	NA
	Yes	90 (65)	48 (35)				
Plastic polymer skin barrier film	No	471 (61)	295 (39)	0.79 (0.47–1.33)	0.380	NA	NA
	Yes	48 (67)	24 (33)				
Zinc oxide adhesive	No	489 (63)	292 (37)	1.50 (0.87–2.58)	0.130	NA	NA
	Yes	30 (53)	27 (47)				
Plastic perforated tape	No	481 (63)	283 (37)	1.61 (0.99–2.59)	0.050	1.66 (1–2.75)	0.04
	Yes	38 (51)	36 (49)				
Hydrogel adhesives	No	470 (62)	286 (38)	1.10 (0.69–1.76)	0.660	NA	NA
	Yes	49 (60)	33 (40)				
Other practices	No	410 (62)	254 (38)	0.96 (0.68–1.35)	0.820	NA	NA
	Yes	109 (63)	65 (37)				
Use of barrier film underneath the adhesive for skin protection	No	218 (60)	146 (40)	0.84 (0.62–1.12)	0.240	NA	NA
	Yes	268 (64)	151 (36)				
Use of adhesive removers when removing tapes	No	141 (54)	119 (46)	0.61 (0.45–0.83)	0.002	0.59 (0.42–0.81)	0.002
	Yes	344 (66)	179 (34)				
Type of adhesive remover used when removing tapes							
Alcohol/organic-based products	No	255 (67)	125 (33)	1.23 (0.82–1.84)	0.290	NA	NA
	Yes	89 (62)	54 (38)				
Oil-based solvents	No	215 (66)	113 (34)	0.97 (0.66–1.41)	0.880	NA	NA
	Yes	129 (66)	66 (34)				
Silicone-based removers	No	248 (65)	131 (35)	0.94 (0.63–1.42)	0.790	NA	NA
	Yes	96 (67)	48 (33)				
Other removers	No	297 (67)	149 (33)	1.27 (0.77–2.09)	0.340	NA	NA
	Yes	47 (61)	30 (39)				
Additional strategies for MARSI prevention							
Remove adhesives slowly using moistened gauze/pad	No	129 (65)	68 (35)	1.22 (0.87–1.70)	0.240	NA	NA
	Yes	390 (61)	251 (39)				
Pull adhesive tapes in a horizontal plane	No	318 (60)	210 (40)	0.82 (0.61–1.09)	0.180	0.76 (0.55–1.05)	0.090
	Yes	201 (65)	109 (35)				
Fold the tape back onto itself while wetting the adhesive-skin interface	No	300 (64)	169 (36)	1.21 (0.91–1.60)	0.170	1.47 (1.07–2.01)	0.010
	Yes	219 (59)	150 (41)				
Other practices	No	501 (61)	314 (39)	0.44 (0.16–1.20)	0.110	NA	NA
	Yes	18 (78)	5 (22)				
Abrasion/friction injuries							
Skin assessments at least every 4 h	No	141 (66)	72 (34)	0.48 (0.34–0.68)	< 0.001	0.48 (0.33–0.71)	< 0.001
	Yes	441 (80)	109 (20)				
Local skin care guideline available	No	161 (72)	62 (28)	0.76 (0.54–1.09)	0.140	NA	NA
	Yes	442 (77)	131 (23)				
Use of alcohol-free skin protectants	No	291 (76)	90 (24)	0.88 (0.60–1.28)	0.520	NA	NA
	Yes	212 (79)	58 (21)				
Strategies for injury prevention							
Frequent vigilance	No	147 (72)	57 (28)	0.77 (0.54–1.11)	0.160	NA	NA
	Yes	487 (77)	147 (23)				
Routinely rotating site of monitoring device	No	86 (67)	43 (33)	0.58 (0.39–0.88)	0.010	NA	NA
	Yes	548 (77)	161 (23)				
Routinely changing body position	No	86 (73)	32 (27)	0.84 (0.54–1.31)	0.440	0.58 (0.34–0.98)	0.040
	Yes	548 (76)	172 (24)				
Use of pressure injury prevention devices	No	360 (75)	118 (25)	0.95 (0.69–1.31)	0.790	NA	NA
	Yes	274 (76)	86 (24)				
Special purpose mattress	No	474 (75)	160 (25)	0.81 (0.55–1.18)	0.280	NA	NA
	Yes	160 (78)	44 (22)				
Petroleum based ointments	No	566 (77)	173 (23)	1.49 (0.94–2.35)	0.080	1.73 (1.06–2.82)	0.020
	Yes	68 (69)	31 (31)				
Availability of a skin assessment tool (local tool or none)	No	91 (64)	52 (36)	0.45 (0.22–0.91)	0.020	NA	NA
	Yes	50 (79)	13 (21)				
Frequency of skin assessment differed for infants ≤ 25 wk	No	389 (76)	121 (24)	1.16 (0.83–1.60)	0.360	NA	NA
	Yes	227 (73)	82 (27)				
Head to toe examination at least 6 h	No	389 (76)	121 (24)	0.83 (0.60–1.16)	0.280	NA	NA
	Yes	261 (77)	77 (23)				
Oil-based emollient application	No	384 (73)	139 (27)	0.71 (0.51–1.00)	0.050	0.65 (0.45–0.95)	0.020
	Yes	250 (79)	65 (21)				
Petrolatum-base emollient application	No	522 (76)	161 (24)	1.24 (0.83–1.80)	0.270	NA	NA
	Yes	112 (72)	43 (28)				
Pressure injuries							
Skin assessments at least every 4 h	No	148 (69)	66 (31)	0.50 (0.35–0.72)	< 0.001	0.51 (0.34–0.78)	0.002
	Yes	452 (82)	102 (18)				
Local skin care guideline available	No	168 (75)	57 (25)	0.71 (0.49–1.03)	0.070	0.71 (0.47–1.09)	0.120
	Yes	463 (80)	113 (20)				
Use of alcohol-free skin protectants	No	293 (76)	90 (24)	0.74 (0.50–1.09)	0.130	NA	NA
	Yes	222 (81)	51 (19)				
Injury prevention strategies							
Frequent vigilance	No	160 (78)	46 (22)	0.95 (0.65–1.39)	0.800	NA	NA
	Yes	500 (78)	137 (22)				
Routinely rotating site of monitoring device	No	102 (79)	27 (21)	1.05 (0.66–1.67)	0.810	NA	NA
	Yes	558 (78)	156 (22)				
Routinely changing body position	No	99 (84)	19 (16)	1.52 (0.90–2.56)	0.110	NA	NA
	Yes	561 (77)	164 (23)				
Use of pressure injury prevention devices	No	380 (79)	100 (21)	1.12 (0.81–1.56)	0.470	NA	NA
	Yes	280 (77)	83 (23)				
Special purpose mattress	No	494 (77)	144 (23)	0.80 (0.54–1.19)	0.280	NA	NA
	Yes	166 (81)	39 (19)				
Petrolatum-based ointments	No	591 (79)	153 (21)	1.67 (1.05–2.67)	0.020	NA	NA
	Yes	69 (70)	30 (30)				
Availability of a skin assessment tool (local tool or none)	No	110 (77)	33 (23)	1.23 (0.62–2.42)	0.540	NA	NA
	Yes	46 (73)	17 (27)				
Frequency of skin assessment differed for infants ≤ 25 wk	No	400 (78)	121 (22)	1.04 (0.74–1.46)	0.810	NA	NA
	Yes	240 (77)	70 (23)				
Head to toe examination at least 6 h	No	363 (77)	111 (23)	0.84 (0.60–1.18)	0.330	NA	NA
	Yes	270 (79)	70 (21)				
Petrolatum-based emollient application	No	550 (80)	136 (20)	1.72 (1.17–2.55)	0.006	1.52 (0.94–2.46)	0.080
	Yes	110 (70)	47 (30)				
Perineal injuries							
Skin assessments at least every 4 h	No	142 (66)	72 (34)	0.58 (0.41–0.82)	0.002	0.52 (0.36–0.75)	0.001
	Yes	425 (77)	126 (23)				
Local skin care guideline available	No	159 (71)	66 (29)	0.76 (0.54–1.07)	0.120	0.66 (0.45–0.96)	0.030
	Yes	436 (76)	138 (24)				
Strategies for injury prevention							
Frequent vigilance	No	160 (77)	47 (23)	1.26 (0.87–1.82)	0.220	NA	NA
	Yes	462 (73)	171 (27)				
Routinely rotating site of monitoring device	No	97 (76)	31 (24)	1.11 (0.71–1.72)	0.620	NA	NA
	Yes	525 (74)	187 (26)				
Routinely changing body position	No	96 (82)	21 (18)	1.71 (1.03–2.82)	0.030	1.94 (0.88–4.25)	0.09
	Yes	526 (73)	197 (27)				
Use of pressure injury prevention devices	No	359 (75)	120 (25)	1.11 (0.81–1.52)	0.490	NA	NA
	Yes	263 (73)	98 (27)				
Special purpose mattress	No	480 (75)	157 (25)	1.31 (0.92–1.86)	0.120	1.33 (0.90–1.97)	0.150
	Yes	142 (70)	61 (30)				
Petrolatum-based ointments	No	558 (73)	183 (25)	1.66 (1.06–2.60)	0.020	1.50 (0.89–2.53)	0.120
	Yes	64 (65)	35 (35)				
Availability of a skin assessment tool (local tool or none)	No	94 (65)	50 (35)	0.87 (0.46–1.64)	0.670	NA	NA
	Yes	43 (68)	20 (32)				
Frequency of skin assessment differed for infants ≤ 25 wk	No	372 (73)	137 (27)	0.89 (0.64–1.24)	0.510	NA	NA
	Yes	233 (75)	77 (25)				
Head to toe examination at least 6 h	No	347 (73)	126 (27)	0.89 (0.64–1.23)	0.480	NA	NA
	Yes	256 (76)	83 (24)				
Oil-base emollient application	No	382 (73)	144 (27)	0.81 (0.59–1.13)	0.220	NA	NA
	Yes	240 (76)	74 (24)				
Petrolatum-base emollient application	No	523 (76)	161 (24)	1.87 (1.29–2.70)	0.001	1.88 (1.21–2.91)	0.004
	Yes	99 (63)	57 (37)				
Diaper dermatitis							
Skin assessments at least every 4 h	No	121 (57)	93 (43)	0.77 (0.56–1.06)	0.110	0.71 (0.51–0.99)	0.040
	Yes	345 (63)	205 (37)				
Local skin care guideline available	No	134 (60)	91 (40)	0.90 (0.65–1.23)	0.530	NA	NA
	Yes	355 (62)	218 (38)				
Strategies to injury prevention							
Frequent vigilance	No	128 (62)	79 (38)	1.07 (0.77–1.47)	0.670	NA	NA
	Yes	381 (60)	252 (40)				
Routinely rotating site of monitoring device	No	81 (62)	49 (38)	1.08 (0.74–1.60)	0.660	NA	NA
	Yes	428 (60)	282 (40)				
Routinely changing body position	No	79 (67)	39 (33)	1.37 (0.91–2.07)	0.120	1.64 (0.92–2.90)	0.080
	Yes	430 (60)	292 (40)				
Use of pressure injury prevention devices	No	288 (60)	191 (40)	0.95 (0.72–1.26)	0.740	NA	NA
	Yes	221 (61)	140 (39)				
Special purpose mattress	No	389 (61)	246 (39)	1.12 (0.81–1.54)	0.480	NA	NA
	Yes	120 (59)	85 (41)				
Petroleum based ointments	No	453 (61)	288 (39)	1.20 (0.79–1.84)	0.380	NA	NA
	Yes	56 (57)	43 (43)				
Availability of a skin assessment tool (local tool or none)	No	88 (61)	56 (39)	1.38 (0.75–2.51)	0.290	NA	NA
	Yes	33 (53)	29 (47)				
Frequency of skin assessment differed for infants ≤ 25 wk	No	303 (59)	208 (41)	0.92 (0.69–1.24)	0.620	NA	NA
	Yes	188 (61)	120 (39)				
Head to toe examination at least 6 h	No	290 (61)	183 (39)	1.08 (0.81–1.43)	0.590	NA	NA
	Yes	201 (59)	137 (41)				
Oil-based emollient application	No	305 (58)	219 (42)	0.76 (0.57–1.02)	0.060	NA	NA
	Yes	204 (65)	112 (35)				
Petrolatum-based emollient application	No	429 (63)	254 (37)	1.62 (1.14–2.30)	0.006	1.62 (1.12–2.33)	0.009
	Yes	80 (51)	77 (49)				
Complications from emollient use							
Increased CONS infection							
Prophylactic application	No	136 (93)	10 (7)	0.43 (0.17–1.10)	0.080	0.38 (0.15–0.99)	0.040
	Yes	279 (97)	9 (3)				
Oil-based emollient	No	178 (94)	12 (6)	0.37 (0.14–0.97)	0.040	NA	NA
	Yes	275 (98)	7 (2)				
Petrolatum-based emollient	No	323 (98)	8 (2)	3.41 (1.34–8.69)	0.010	3.66 (1.42–9.46)	0.007
	Yes	130 (92)	11 (8)				
Hyperthermia							
Prophylactic application	No	135 (96)	5 (4)	0.87 (0.28–2.64)	0.800	NA	NA
	Yes	279 (97)	9 (3)				
Oil-based emollient	No	177 (96)	7 (4)	1.02 (0.39–2.69)	0.950	NA	NA
	Yes	271 (96)	11 (4)				
Petrolatum-based emollient	No	320 (98)	7 (2)	3.92 (1.48–10.35)	0.006	NA	NA
	Yes	128 (92)	11 (8)				
Tissue burns							
Prophylactic application	No	137 (97)	4 (3)	1.36 (0.42–4.34)	0.600	NA	NA
	Yes	277 (96)	11 (4)				
Oil-based emollient	No	178 (96)	7 (4)	0.75 (0.26–2.10)	0.580	NA	NA
	Yes	271 (97)	8 (3)				
Petrolatum-based emollient	No	318 (98)	6 (2)	3.64 (1.27–10.43)	0.010	NA	NA
	Yes	131 (94)	9 (6)				
Interference with adhesive tapes							
Prophylactic application	No	113 (78)	31 (26)	1.10 (0.68–1.79)	0.680	NA	NA
	Yes	221 (77)	67 (23)				
Oil-based emollient	No	143 (77)	42 (23)	1.12 (0.73–1.74)	0.580	NA	NA
	Yes	214 (75)	71 (25)				
Petrolatum-based emollient	No	259 (79)	70 (21)	1.62 (1.04–2.52)	0.030	NA	NA
	Yes	98 (70)	43 (31)				
Environmental contamination leading to invasive sepsis							
Prophylactic application	No	133 (96)	6 (4)	1.13 (0.42–3.02)	0.790	NA	NA
	Yes	273 (95)	14 (3)				
Oil-based emollient	No	173 (95)	9 (5)	1.00 (0.42–2.37)	0.990	NA	NA
	Yes	269 (95)	14 (5)				
Petrolatum-based emollient	No	318 (97)	9 (3)	4.02 (1.69–9.53)	0.002	NA	NA
	Yes	123 (90)	14 (10)				

Responses reported as number (%), percentages rounded to the nearest whole number. Adjusted odds ratio from stepwise backward binary multivariate logistic regression models, adjusted for regions. Uncommon occurrence of skin injuries was arbitrarily used as the reference group (base). Occurrence of injuries uncommon: responses rare and seldom; occurrence of injuries common: responses often, almost always and always. *MARSI* medical adhesive-related skin injury, *CONS* coagulase negative staphylococci, *OR* unadjusted odds ratio, *aOR* adjusted odds ratio, *CI* confidence interval, *NA* effect output not included as *P* ≥ 0.2 for stepwise regression

## Results

Responses from 848 NICUs from six geographic regions (Europe, Asia, North America, Africa, South America, and Oceania) and from low and lower middle-income countries (low and LMIC), upper middle-income countries (UMIC) and high-income countries (HIC) were received. The World Bank assigns each country one of the four groups: low, lower middle, upper middle, and high-income countries based on its economic performance. We used the World Bank report for 2021 to reflect the income status category of the participating unit’s country.

### Skin injuries

**Fig. 1:**
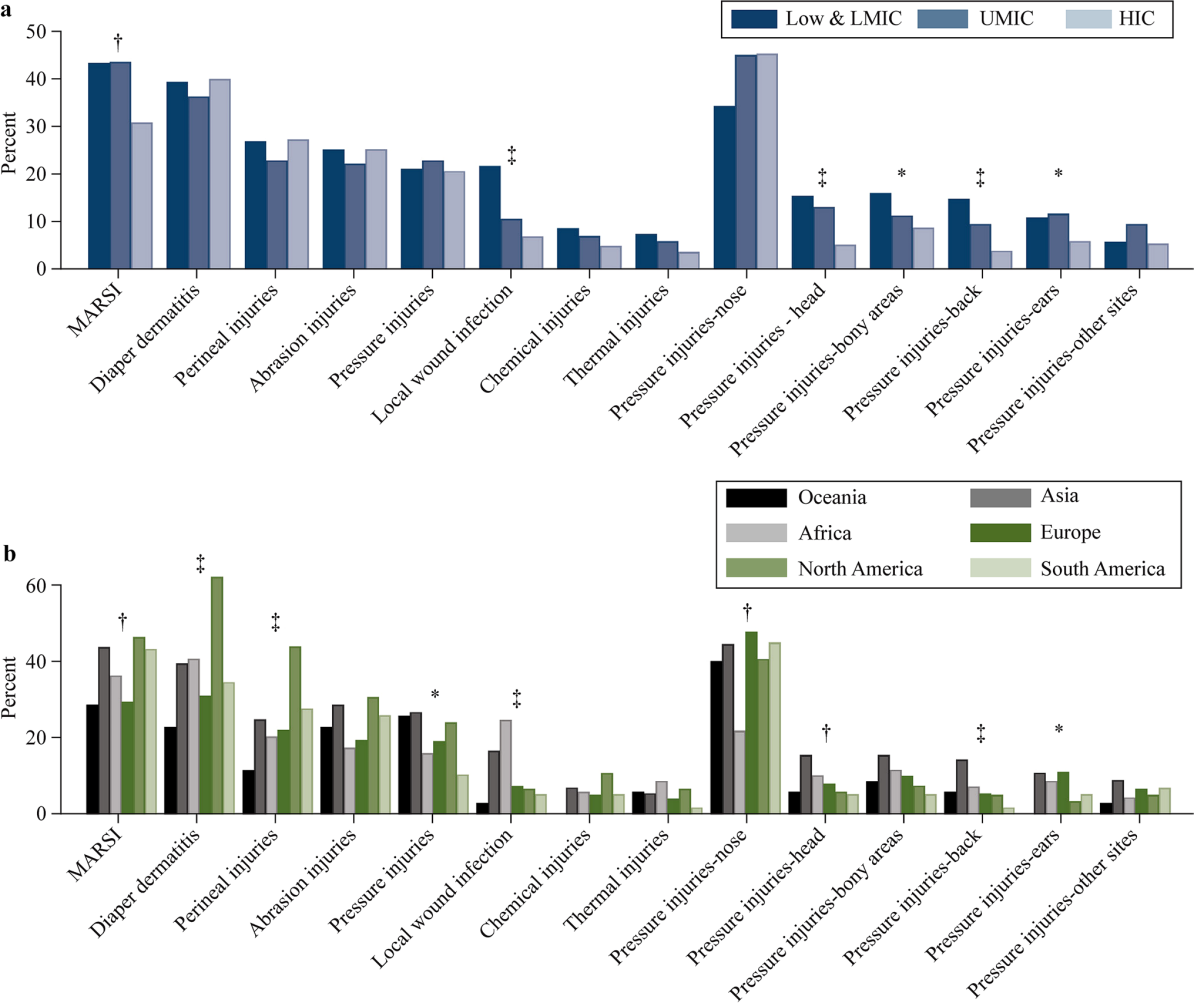
Occurrence of skin injuries based on income status group (**a**) and geographic region (**b**). *MARSI* medical adhesive-related skin injury, *LMIC* lower middle-income countries, *UMIC* upper middle-income countries, *HIC* high-income countries. ^*^P ≥ 0.01 and < 0.05, ^†^*P* ≥ 0.001 and < 0.01, ^‡^*P* < 0.001

Diaper dermatitis (331/840, 39%) and medical adhesive-related skin injury (MARSI) (319/838, 38%) were the most common injuries, followed by perineal (218/840, 26%), abrasion (204/838, 24%), pressure injuries (183/843, 22%), and local infection (94/840, 11%). Diaper dermatitis differed between geographic regions (Fig. 1). The odds of diaper dermatitis were higher in NICUs from Asia (OR = 1.45, 95% CI = 1.02–2.06; *P* = 0.03) and North America (OR = 3.77, 95% CI = 2.51–5.89; *P* < 0.001) compared to European NICUs, and in NICUs applying petrolatum-based emollient (aOR = 1.62, 95% CI = 1.12–2.33; *P* = 0.009).

**Fig. 2:**
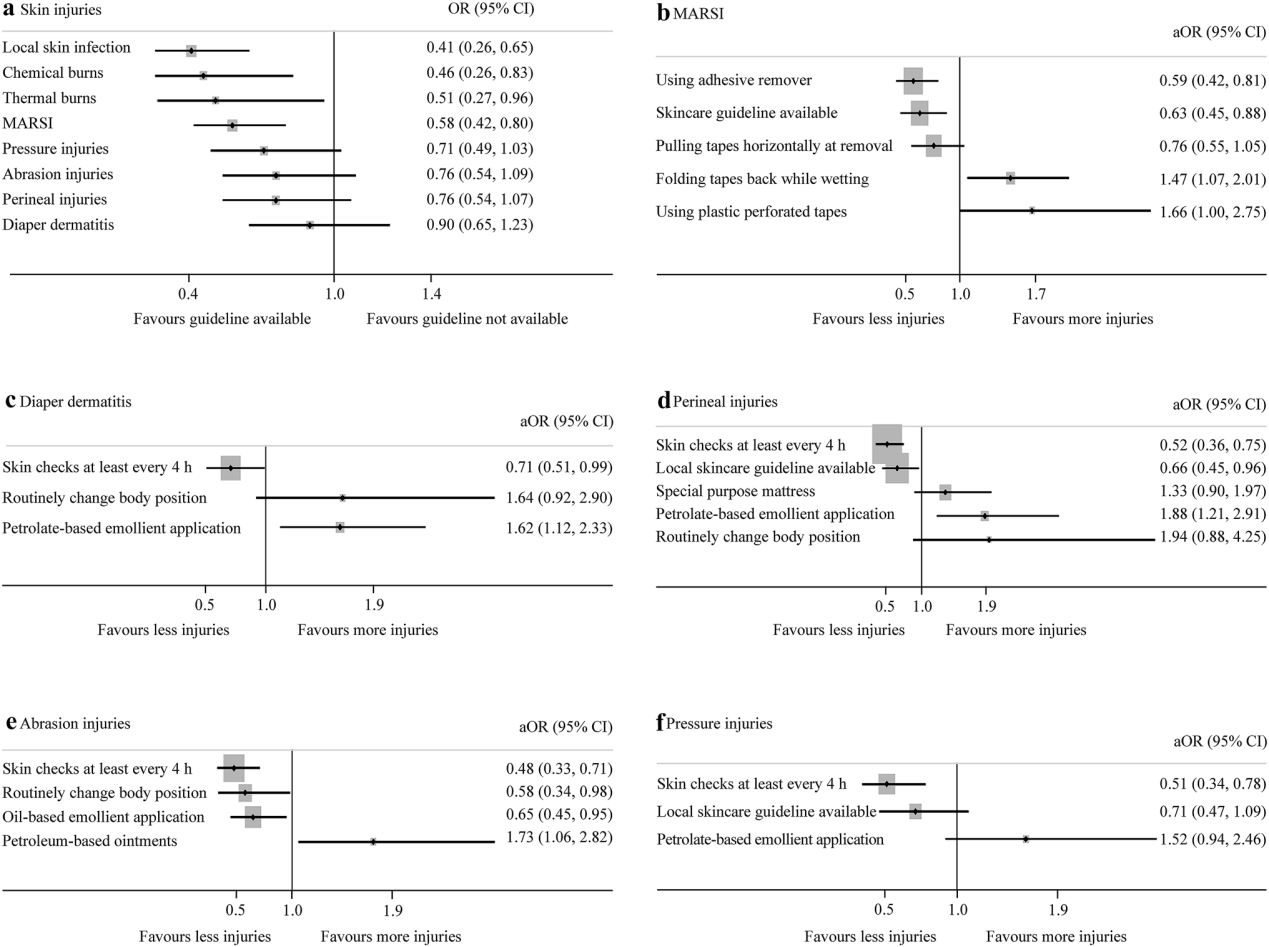
Relationship between practices and the odds of skin injuries. **a** Availability of skincare guideline and odds of skin injuries (from univariable models); **b** practices and odds of MARSI (from multivariable model); **c** practices and odds of diaper dermatitis (from multivariable model); **d** practices and odds of perineal injuires (from multivariable model); **e** practices and odds of abrasion injuries (from multivariable model); **f** practices and odds of pressure injuries (from multivariable model). *MARSI* medical adhesive-related skin injury, *CI* confidence interval, *OR* unadjusted odds ratio from univariable model, *aOR* adjusted odds ratio from multivariable model

Having a local skincare guideline (aOR = 0.63, 95% CI = 0.45–0.88; *P* = 0.008) and using adhesive tape removers (aOR = 0.59, 95% CI = 0.42–0.81; *P* = 0.002) reduced the odds of MARSI. The odds of MARSI were higher in NICUs using plastic perforated tapes (aOR = 1.66, 95% CI = 1.00–2.75; *P* = 0.04) for securing tubes and folding the adhesive tape backwards and wetting it during its removal (aOR = 1.47, 95% CI = 1.07–2.01; *P* = 0.01) (Fig. 2). The odds were lower in NICUs from HIC (OR = 0.56, 95% CI = 0.39–0.81; *P* = 0.002) compared to NICUs from low and LMIC, and UMIC. The odds were higher in NICUs from Asia (OR = 1.91, 95% CI = 1.34–2.71; *P* < 0.001), North America (OR = 2.08, 95% CI = 1.34–3.23; *P* = 0.001) and South America (OR = 1.86, 95% CI = 1.04–3.32; *P* = 0.03) compared to European NICUs.

The odds of perineal injuries were higher in NICUs from North America (OR = 2.71, 95% CI = 1.72–4.26; *P* < 0.001) compared to European NICUs. The odds were lower when skin assessments were performed at least every four hours (aOR = 0.52, 95% CI = 0.36–0.75; *P* = 0.001) and when a local skincare guideline was available (aOR = 0.66, 95% CI = 0.45–0.96; *P* = 0.03). The odds of perineal injuries were higher when petrolatum-based emollients were applied (aOR = 1.88, 95% CI = 1.21–2.91; *P* = 0.004) (Fig. 2).

Abrasion injuries were reported most from North America (37/121, 31%) and least from Africa (12/68, 18%) (Fig. 1). The odds were lower when NICUs performed skin assessments at least every four hours (aOR = 0.48, 95% CI = 0.33–0.71; *P* < 0.001), applied oil-based emollients (aOR = 0.65, 95% CI = 0.45–0.95; *P* = 0.02), routinely changed the body position (aOR = 0.58, 95% CI = 0.34–0.98; *P* = 0.04), routinely rotated the device site (OR = 0.58, 95% CI = 0.39–0.88; *P* = 0.01) and used a locally developed skin assessment tool (as compared to none; OR = 0.45, 95% CI = 0.22–0.961; *P* = 0.02) (Fig. 2 and Table 1). The odds were higher when applying petrolatum-based ointments (aOR = 1.73, 95% CI = 1.06–2.82; *P* = 0.02) and in NICUs from Asia (OR = 1.67, 95% CI = 0.54–2.93; *P* = 0.01) and North America (OR = 1.8, 95% CI = 1.11–2.92; *P* = 0.01) compared to European NICUs.

Nasal pressure injuries were most common (363/797, 46%) (Fig. 1). The odds were higher in NICUs from Asia (OR = 1.55, 95% CI = 1.04–2.31; *P* = 0.03) compared to European NICUs. The odds were lower when NICUs assessed the sites at least every four hours (aOR = 0.51, 95% CI = 0.34–0.78; *P* = 0.002).

### Skincare practices

Local skincare and skin antisepsis guidelines were available for 72% (579/805) and 75% (605/811) NICUs respectively. Availability differed between income status groups and geographic regions (Tables 2 and 3). Skin injuries were lower when a local skincare guideline was available (Fig. 2).

**Table 2:** Practices based on income status groups of the respondent units

Practices	Low and LMIC (175/842, 21%)	UMIC (275/842, 33%)	HIC (392/842, 47%)	Level of association, *P*
Local skin care guideline available (*n* = 799)	100/167 (60)	190/260 (73)	284/372 (76)	< 0.001
Local skin antisepsis guideline available (*n* = 805)	99/170 (58)	195/260 (75)	306/375 (82)	< 0.001
Skin cleansing solution prior to sterile procedures (*n* = 842)^a^	*n* = 175	*n* = 275	*n* = 392	< 0.001
Aqueous chlorhexidine solution	50 (29)	93 (34)	211 (54)	
Combination alcohol and antiseptic	106 (61)	72 (26)	107 (27)	
Iodine-based solution	72 (41)	146 (53)	93 (24)	
Hexachlorophene	2 (1)	3 (1)	8 (2)	
Sterile water	12 (7)	28 (10)	50 (13)	
Skin cleansing solution prior to clean procedures (*n* = 828)^a^	*n* = 172	*n* = 271	*n* = 385	< 0.001
Aqueous chlorhexidine solution	15 (9)	44 (16)	106 (28)	
Combination alcohol and antiseptic	129 (75)	172 (63)	180 (47)	
Iodine-based solution	10 (6)	34 (13)	9 (2)	
Hexachlorophene	1 (1)	2 (1)	3 (1)	
Sterile water	7 (4)	4 (1)	16 (4)	
Others^b^	10 (6)	15 (6)	71 (18)	
Differing skin antisepsis for infants ≤ 25 wk GA (*n* = 833)^c^	24/172 (14)	60/273 (22)	119/388 (31)	< 0.001
Skin integrity assessment tool (*n* = 842)^a^	*n* = 175	*n* = 275	*n* = 392	< 0.001
Braden Q	6 (3)	70 (25)	39 (10)	
Neonatal skin risk assessment tool	66 (38)	57 (21)	61 (16)	
Neonatal skin condition score	26 (15)	55 (20)	56 (14)	
Starkid skin scale	1 (1)	5 (2)	1 (-)	
Neonatal skin risk assessment scale	12 (7)	65 (24)	34 (9)	
Glamorgan pressure injury risk assessment	0 (0)	9 (3)	12 (3)	
Other local tools^d^	4 (2)	9 (3)	49 (13)	
None	40 (23)	32 (12)	74 (19)	
Umbilical cord care practices (*n* = 842)^a^	*n* = 175	*n* = 275	*n* = 392	< 0.001
Leave alone	105 (60)	162 (59)	283 (72)	
Sterile water	46 (26)	33 (12)	50 (13)	
A drying agent	20 (11)	57 (21)	25 (6)	
Topical antibiotic agent	8 (5)	25 (9)	11 (3)	
Topical antifungal agent	2 (1)	5 (2)	1 (-)	
Topical breast milk	2 (1)	7 (3)	0 (0)	
Others^e^	16 (9)	29 (11)	44 (11)	
Routine use of topical emollients (*n* = 805), of these 41 (5%) were used for specific GA infants	96/167 (57)	135/258 (52)	104/380 (27)	< 0.001
Frequency of emollient use (*n* = 334)	*n* = 95	*n* = 136	*n* = 103	0.004
Once daily	37 (39)	63 (46)	38 (37)	
Twice daily	41 (43)	29 (21)	32 (31)	
More than twice daily	13 (14)	31 (23)	17 (16)	
Others	4 (4)	13 (10)	16 (16)	
Type of topical emollient used^a^ (*n* = 842), not just prophylactic	*n* = 175	*n* = 275	*n* = 392	< 0.001
Oil-based	99 (57)	123 (45)	95 (24)	
Petrolatum-based	27 (15)	70 (25)	59 (15)	
Others^f^	6 (3)	32 (12)	52 (13)	
Issues (often, almost always and always) from any use of emollients^a^				
Interference with other adhesives (*n* = 469)	27/110 (25)	47/178 (26)	39/181 (22)	0.550
Increased incidence of CONS infection (*n* = 471)	4/109 (4)	8/179 (4)	7/183 (4)	0.930
Hyperthermia (*n* = 465)	4/110 (4)	11/176 (6)	3/179 (2)	0.080
Tissue burns (*n* = 463)	6/110 (5)	5/176 (3)	4/177 (2)	0.300
Environmental contamination causing invasive sepsis (*n* = 463)	9/109 (8)	11/176 (6)	3/178 (2)	0.020
MARSI prevention				
Tapes for securing tubes (*n* = 848)^a^	*n* = 175	*n* = 275	*n* = 392	0.001
Transparent film dressing	70 (40)	116 (42)	131 (33)	
Hydrocolloid base with transparent film or adhesive tape	30 (17)	98 (36)	154 (39)	
Silicone tape	32 (18)	39 (14)	68 (17)	
Plastic polymer skin barrier film	9 (5)	34 (12)	29 (7)	
Zinc oxide adhesive	26 (15)	16 (6)	16 (4)	
Plastic perforated tape	10 (6)	26 (10)	39 (10)	
Hydrogel adhesive	8 (5)	25 (9)	49 (13)	
Others^g^	27 (15)	54 (20)	94 (24)	
Use of barrier film underneath the adhesive for skin protection (*n* = 787)	69/162 (43)	132/254 (52)	220/371 (59)	0.002
Use of adhesive remover when removing tapes (*n* = 787)	56/162 (35)	170/257 (66)	298/368 (81)	< 0.001
Type of adhesive remover used (*n* = 524 as 4 did not identify their country)^a^	*n* = 56	*n* = 170	*n* = 298	< 0.001
Alcohol/organic-based products	31 (55)	58 (34)	55 (19)	
Oil-based solvents	22 (39)	80 (47)	92 (31)	
Silicone-based removers	3 (5)	45 (27)	96 (32)	
Others^h^	5 (9)	17 (10)	56 (19)	
Additional strategy for MARSI prevention (*n* = 842)^a^	*n* = 175	*n* = 275	*n* = 392	< 0.001
Remove adhesive slowly and carefully using moistened gauze/pad	121 (69)	212 (77)	312 (80)	
Pull adhesive tape in a horizontal plane	55 (31)	114 (42)	144 (37)	
Fold the tape back onto itself while continuously wetting the adhesive-skin interface	56 (32)	138 (50)	177 (45)	
Others^i^	4 (2)	5 (2)	14 (4)	

Six respondents did not identify their country. Responses reported as number (%), percentage rounded to the nearest whole number. *LMIC* lower middle-income country, *UMIC* upper middle-income country, *HIC* high-income country, *GA* gestational age, *MARSI* medical adhesive-related skin injury, *CONS* coagulase-negative *Staphylococcal*. ^a^Multiple responses allowed; ^b^other solutions were alcohol, chlorine, chlorhexidine/alcohol and benzalkonium, sodium chloride, octenidine and hypochlorite; ^c^other practices such as use of only sterile water, povidone-iodine, weak non-alcoholic solution, octenidine with sterile water and wiping off the cleansing solution with sterile water; ^d^visual inspection, homegrown local tool, neonatal skin injury and pressure injury risk assessment, Swiss neonatal skin score, Norton pressure sore risk; ^e^other topical cord application practices included application of varying strengths of chlorhexidine or alcohol-based solutions, normal saline, calendula tincture, hydrogen peroxide, iodine-based solutions, octinidine solution, methylated spirits, and use of soap and water; ^f^baby oil, benzalkonium, ceramide base, cold cream, dimethacone, eucerin, silicone ointment, oil with vitamin E; ^g^adhesive paper or plaster, band aid, brown tape, cotton or cloth tape, polyacrylate tape, silk tape; ^h^coconut oil, water, soap and water, emollient, saline, octenidine dihydrochloride and 2-phenoxyethanol; ^i^keep adhesive tapes for 24 hours, olive oil moistened cotton wool, avoid band aids, loosen edges of tape with adhesive remover and carefully peel back dressing until it is removed followed by clean site with saline wipe

**Table 3:** Practices based on geographic region of the respondent units

Practices	Europe (300/848)	Asia (259/848)	North America (121/848)	Africa (69/848)	South America (58/848)	Oceania (35/848)	Level of association, *P*
Local skin care guideline available (*n* = 799)	213/288 (74)	159/240 (66)	89/115 (77)	42/67 (63)	46/55 (84)	25/34 (74)	0.020
Local skin antisepsis guideline available (*n* = 805)	231/287 (80)	157/244 (64)	88/116 (76)	42/67 (63)	52/57 (91)	30/34 (88)	< 0.001
Skin cleansing solution prior to sterile procedures (*n* = 842)^a^	*n* = 300	*n* = 259	*n* = 121	*n* = 69	*n* = 58	*n* = 35	< 0.001
Aqueous chlorhexidine solution^b^	109 (36)	96 (37)	56 (46)	16 (23)	46 (79)	31 (89)	
Combination alcohol and antiseptic	98 (33)	93 (36)	27 (22)	49 (71)	13 (22)	5 (14)	
Iodine-based solution	1000 (33)	122 (47)	63 (52)	24 (35)	1 (2)	1 (3)	
Hexachlorophene	3 (1)	8 (3)	2 (2)	0 (0)	0 (0)	0 (0)	
Sterile water	37 (12)	24 (9)	17 (14)	6 (9)	4 (7)	2 (6)	
Skin cleansing solution prior to clean procedures (*n* = 828)^a^	*n* = 295	*n* = 254	*n* = 120	*n* = 67	*n* = 57	*n* = 35	< 0.001
Aqueous chlorhexidine solution	66 (22)	36 (14)	24 (20)	0 (0)	28 (49)	11 (31)	
Combination alcohol and antiseptic	166 (66)	162 (64)	67 (56)	53 (79)	22 (39)	11 (31)	
Iodine-based solution	13 (4)	29 (11)	6 (5)	4 (6)	1 (2)	0 (0)	
Hexachlorophene	1 (-)	5 (2)	0 (0)	0 (0)	0 (0)	0 (0)	
Sterile water	10 (3)	5 (2)	2 (2)	4 (6)	1 (2)	5 (14)	
Others	39 (13)	17 (7)	21 (18)	6 (9)	5 (9)	8 (23)	
Differing skin antisepsis for infants ≤ 25 wk GA (*n* = 833)	78/297 (26)	57/258 (22)	40/120 (33)	4/67 (6)	12/57 (21)	12/34 (35)	0.001
Skin integrity assessment tool (*n* = 842)^a^	*n* = 300	*n* = 259	*n* = 121	*n* = 69	*n* = 58	*n* = 35	< 0.001
Braden Q	48 (16)	38 (15)	16 (13)	1 (1)	10 (17)	2 (6)	
Neonatal skin risk assessment tool	53 (18)	58 (22)	17 (14)	34 (49)	12 (21)	10 (29)	
Neonatal skin condition score	43 (14)	49 (19)	25 (21)	5 (7)	5 (9)	10 (29)	
Starkid skin scale	1 (-)	5 (2)	1 (1)	0 (0)	0 (0)	0 (0)	
Neonatal skin risk assessment scale	41 (14)	44 (17)	12 (10)	2 (3)	7 (12)	5 (14)	
Glamorgan pressure injury risk assessment	6 (2)	4 (2)	3 (2)	0 (0)	0 (0)	8 (23)	
Other local tools	28 (9)	13 (5)	14 (12)	0 (0)	2 (3)	5 (14)	
None	54 (18)	38 (15)	22 (18)	15 (22)	13 (3)	4 (11)	
Umbilical cord care practices (*n* = 842)^a^	*n* = 300	*n* = 259	*n* = 121	*n* = 69	*n* = 58	*n* = 35	< 0.001
Leave alone	221 (74)	158 (61)	100 (83)	13 (19)	26 (45)	32 (91)	
Sterile water	41 (14)	34 (13)	9 (7)	34 (49)	8 (14)	3 (9)	
Drying agent	26 (9)	37 (14)	6 (5)	13 (19)	20 (34)	0 (0)	
Topical antibiotic agent	10 (3)	25 (10)	4 (3)	5 (7)	0 (0)	0 (0)	
Topical antifungal agent	1 (-)	6 (2)	1 (1)	0 (0)	0 (0)	0 (0)	
Topical breast milk	0 (0)	6 (2)	0 (0)	1 (1)	2 (3)	0 (0)	
Others	26 (9)	32 (12)	6 (5)	14 (20)	9 (16)	2 (6)	
Routine use of topical emollients (*n* = 805), of these 41 (5%) were used for specific GA infants	131/290 (45)	106/242 (44)	36/120 (30)	41/65 (63)	14/54 (26)	7/34 (20)	< 0.001
Frequency of emollient use (*n* = 334)	*n* = 131	*n* = 105	*n* = 36	*n* = 41	*n* = 14	*n* = 7	0.002
Once daily	53 (40)	49 (47)	12 (33)	12 (29)	10 (71)	2 (29)	
Twice daily	36 (27)	28 (27)	12 (33)	24 (59)	0 (0)	2 (29)	
More than twice daily	23 (18)	22 (21)	7 (19)	5 (12)	3 (21)	1 (14)	
Others	19 (15)	6 (6)	5 (14)	0 (0)	1 (7)	2 (29)	
Type of topical emollient used (*n* = 842)^a^ not just prophylactic	*n* = 300	*n* = 259	*n* = 121	*n* = 69	*n* = 58	*n* = 35	< 0.001
Oil-based	127 (42)	107 (41)	21 (17)	45 (65)	15 (26)	2 (6)	
Petrolatum-based	69 (23)	47 (18)	24 (20)	9 (13)	4 (7)	3 (9)	
Others	37 (12)	14 (5)	21 (17)	4 (6)	7 (12)	7(20)	
Issues (often, almost always and always) from any use of emollients^a^							
Interference with other adhesives (*n* = 469)	46/196 (23)	37/132 (28)	13/59 (22)	10/51 (20)	4/18 (22)	3/13 (23)	0.880
Increased incidence of CONS infection (*n* = 471)	3/196 (2)	10/134 (7)	4/60 (7)	2/50 (4)	0/18 (0)	0/13 (0)	0.080
Hyperthermia (*n* = 465)	8/193 (4)	5/134 (4)	2/57 (4)	3/50 (6)	0/18 (0)	0/13 (0)	0.950
Tissue burns (*n* = 463)	2/191 (1)	10/133 (8)	1/58 (2)	2/50 (4)	0/18 (0)	0/13 (0)	0.370
Environmental contamination causing invasive sepsis (*n* = 463)	5/192 (3)	12/132 (9)	3/58 (5)	3/50 (6)	0/18 (0)	0/13 (0)	0.150
MARSI prevention							
Tapes for securing tubes (*n* = 842)^a^	*n* = 300	*n* = 259	*n* = 121	*n* = 69	*n* = 58	*n* = 35	< 0.001
Transparent film dressing	100 (33)	117 (45)	68 (56)	16 (23)	15 (26)	1 (3)	
Hydrocolloid base with transparent film or adhesive tape	91 (30)	76 (29)	52 (43)	11 (16)	34 (59)	18 (51)	
Silicone tape	39 (13)	46 (18)	22 (18)	24 (35)	1 (2)	7 (20)	
Plastic polymer skin barrier film	33 (11)	29 (11)	8 (7)	2 (3)	0 (0)	0 (0)	
Zinc oxide adhesive	170 (6)	13 (5)	2 (2)	21 (30)	1 (2)	4 (11)	
Plastic perforated tape	38 (13)	19 (7)	13 (11)	2 (3)	1 (2)	2 (6)	
Hydrogel adhesive	32 (11)	28 (11)	13 (11)	1 (2)	3 (5)	5 (14)	
Other methods	76 (25)	42 (16)	27 (22)	7 (10)	16 (28)	7 (20)	
Use of barrier film underneath the adhesive (*n* = 787)	143/286 (50)	132/233 (57)	70/117 (60)	22/65 (34)	34/53 (64)	20/33 (61)	0.004
Use of adhesive removers when removing tapes (*n* = 787)	236/286 (83)	141/235 (60)	80/114 (70)	12/64 (19)	31/55 (56)	24/33 (73)	< 0.001
Type of adhesive remover used (*n* = 524, country unknown for 4)^a^	*n* = 226	*n* = 141	*n* = 80	*n* = 12	*n* = 31	*n* = 24	< 0.001
Alcohol/organic-based product	52 (22)	59 (42)	23 (29)	3 (25)	4 (13)	3 (13)	
Oil-based solvent	75 (32)	64 (45)	25 (31)	9 (75)	17 (55)	4 (17)	
Silicone-based remover	81 (34)	19 (14)	27 (34)	0 (0)	6 (19)	11 (46)	
Other agent	43 (18)	14 (10)	9 (11)	1 (8)	5 (16)	6 (25)	
Additional strategy for MARSI prevention (*n* = 842)^a^	*n* = 300	*n* = 259	*n* = 121	*n* = 69	*n* = 58	*n* = 35	< 0.001
Remove adhesives slowly using moistened gauze	243 (81)	201 (78)	95 (79)	36 (52)	39 (67)	31 (89)	
Pull adhesive tapes in a horizontal plane	109 (36)	83 (32)	57 (47)	31 (45)	19 (33)	14 (40)	
Fold the tape back onto itself while continuously wetting the adhesive-skin interface	146 (49)	109 (42)	52 (43)	12 (17)	32 (55)	20 (57)	
Other methods	13 (4)	3 (1)	6 (5)	1 (2)	0 (0)	0 (0)	

Responses reported as number (%), percentage rounded to the nearest whole number. *GA* gestational age, *CONS* coagulase negative staphylococci, *MARSI* medical adhesive-related skin injury. ^a^Multiple responses allowed; ^b^the strength of the chlorhexidine solution varied from 0.01% to 100%

Aqueous chlorhexidine (355/848, 42%), iodine-based solution (314/848, 37%), and a combination of alcohol and antiseptic (286/848, 34%) were the most common skin cleansing agents used prior to sterile procedures. Choice of topical cleansing agent differed based on resource settings (Table 2) and geographic region (Table 3). For skin cleansing prior to clean procedures, 58% (484/834) NICUs used a combination of alcohol and antiseptic solution. NICUs from South America used aqueous chlorhexidine the most (Table 3). A quarter of NICUs followed a differing practice on skin antisepsis for infants ≤ 25 weeks gestation. This practice varied widely across income status groups and regions (Tables 2 and 3). Most NICUs applied nothing to the umbilical cord (553/848, 65%). Sterile water (130/848, 15%) and a drying agent (104/848, 12%) were the most common topical agents used. Umbilical cord practices differed between income status groups and between geographic regions (Tables 2 and 3).

**Fig. 3:**
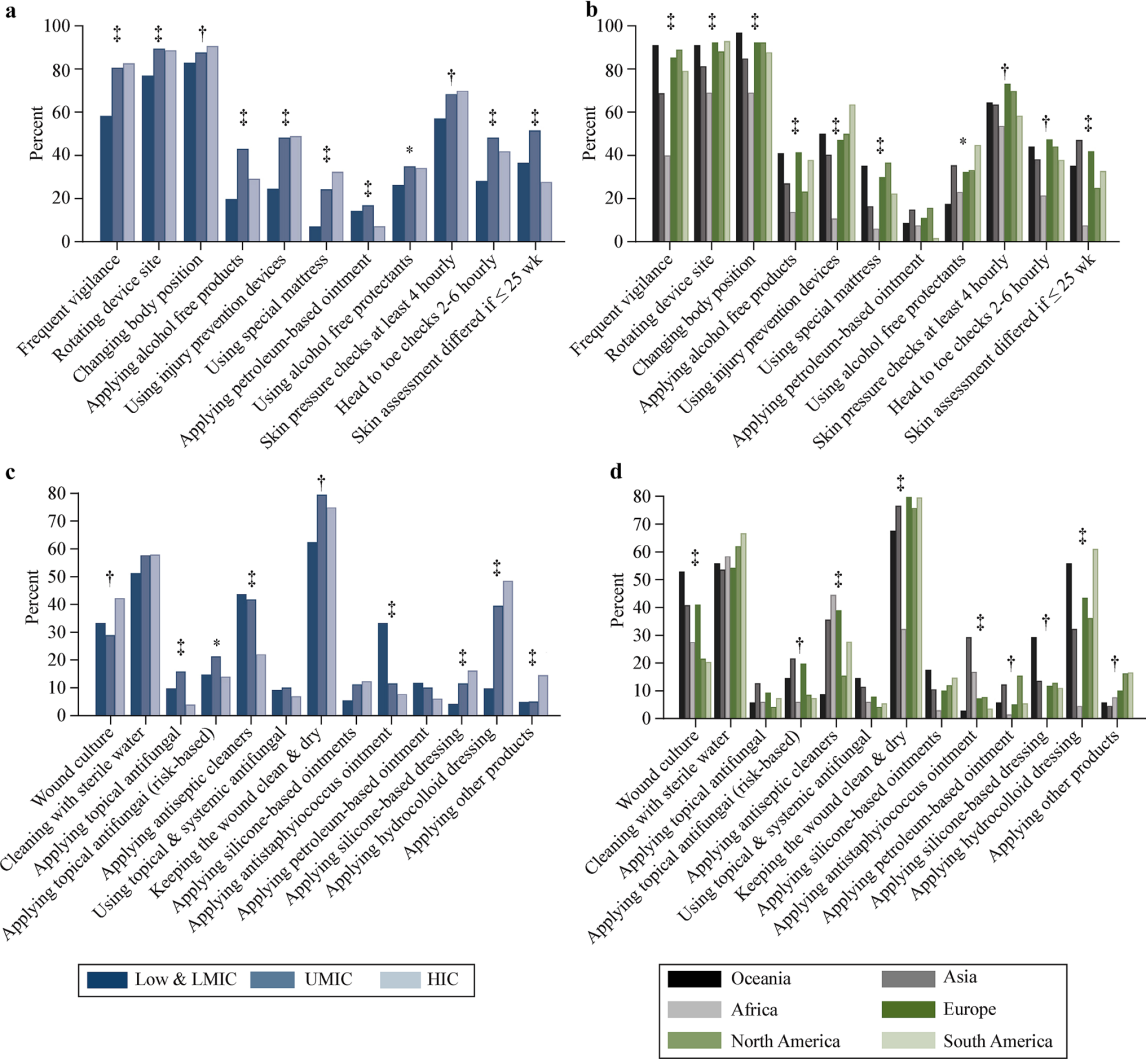
Skin injury prevention and management strategies. **a** Responses by income status group; **b** prevention responses by geographic region; **c** management responses by income status group; **d** management responses by geographic region. Other management practices included application of other products such as zinc-based paste, alginate, artificial skin, *Leptospermum* or medical grade honey and other alternative medicine practices. *LMIC* lower middle-income countries, *UMIC* upper middle-income countries, *HIC* high-income countries, ^*^P ≥ 0.01 and < 0.05, ^†^*P* ≥ 0.001 and < 0.01*, *^‡^*P* < 0.001

A neonatal skin risk assessment tool (184/848, 22%), neonatal skin condition score (140/848, 17%) and the Braden Q scale (116/848, 14%) were the most common skin integrity assessment tools used. No tool was used in 17% (146/848) of NICUs and 7% (62/848) used a locally developed tool. Income group and region-based use of these tools are shown in Tables 2 and 3. There was no relationship between any skin integrity assessment tool and the occurrence of skin injuries, except for the Braden Q tool for perineal injuries (OR = 0.52, 95% CI = 0.31–0.87; *P* = 0.01). Most NICUs (556/771, 72%) were assessing the skin at least every four hours, however, only 60% of NICUs from low and LMIC were performing these assessments at least every four hours compared to NICUs from UMIC and HIC (each 75%) (Fig. 3). European and North American NICUs performed this surveillance more than NICUs from other regions. Changing body position (729/848, 86%), rotating the sites of monitoring devices (716/848, 84%) and frequent site surveillance (639/848, 75%) were the three most common practices used to minimize skin injuries. These were more commonly practiced in NICUs from UMIC and HIC (Fig. 3).

A transparent (318/848, 38%) or a hydrocolloid-based (283/848, 33%) dressing was mostly used for securing tubes to the skin. These practices were similar between income status groups and geographic regions except for NICUs from Africa (Tables 2 and 3). Just over half of the NICUs were using a barrier film underneath the adhesive for MARSI prevention and 67% (528/792) of NICUs were using adhesive removers when removing tapes. Other MARSI prevention practices included removing adhesives carefully using moistened gauze (649/848, 77%), folding the tape back onto itself while continuously wetting the adhesive-skin interface (373/848, 44%) and pulling off the adhesive tapes horizontally (315/848, 37%).

Keeping a wound clean and dry (589/848, 70%), thorough cleaning with sterile water (449/848, 53%), use of hydrocolloid dressings (298/848, 35%), performing surveillance wound cultures (290/848, 34%) and use of antiseptic cleansers (264/848, 31%) were the most common wound practices. Applying antiseptic cleaners and anti-staphylococcus ointments were more common in NICUs from low and LMIC than in NICUs from UMIC and HIC, and geographic variation for these practices was observed (Fig. 3).

Emollients were used prophylactically by 41% (336/810) NICUs; of these 5% used them for specific gestational ages. They were usually applied either once daily (138/335, 41%) or twice daily (102/335, 30%). Applying an oil-based emollient (318/848, 38%) was more common than applying a petrolatum-based emollient (158/848, 19%). Interference with adhesives was the most common complication (113/470, 24%), the occurrence of other complications was low (< 5%). Emollient use was lower in NICUs from HIC compared to NICUs from other two income groups (Table 2). Its use was lower in NICUs from Oceania, South and North America (Table 3). Petrolatum-based emollient was associated with higher odds of complications [coagulase negative staphylococcus infection (aOR = 3.66, 95% CI = 1.42–9.46; *P* = 0.007); hyperthermia (OR = 3.92, 95% CI = 1.48–10.35; *P* = 0.006); tissue burns (OR = 3.64, 95% CI = 1.27–10.43; *P* = 0.01); interference with adhesives (OR = 1.62, 95% CI = 1.04–2.52; *P* = 0.03) and environmental contamination (OR = 4.02, 95% CI = 1.69–9.53; *P* = 0.002)], oil-based emollient which was associated with lower odds of coagulase-negative staphylococcus infection (OR = 0.37, 95% CI = 0.14–0.97; *P* = 0.04).

## Discussion

In this large global survey, skin injuries were common in EP infants. Skin injuries were less when NICUs had a local skincare guideline and performed skin assessments at least every four hours. Geographic region and resource settings-based variation for skin injuries and skincare practices were observed. The reasons for this variation (such as limitations from cost or skills shortage) needs further exploration.

EP infants are at high risk of developing skin injuries [7]. MARSI may occur through various mechanisms [5, 8]. Two common practices included applying a transparent adhesive tape to the skin and applying a hydrocolloid tape in between a transparent tape and the skin. Although hydrogel-based adhesives when removed are gentler on the skin, they were used infrequently by the respondents [9]. While some researchers found certain products or practices reduced MARSI, others reported no effect [9–13]. Evidence is needed regarding which adhesive best secures medical devices and causes the least skin injury. Barrier films protect preterm infants’ skin [14]. At least half of the NICUs were using a barrier film for skin protection. The use of adhesive removers could reduce MARSI when removing tapes, though their efficacy and safety in preterm infants has been questioned [15–17]. MARSI was less in NICUs that followed a local skincare guideline and used an adhesive remover when removing tapes. MARSI can occur with zinc-based adhesives or plastic perforated tapes [9, 18]. Altogether, MARSI was frequent in NICUs from North America, South America and Asia. These NICUs used plastic perforated tapes, which may have contributed to MARSI. Diaper dermatitis is common in term infants [19]. In our survey, diaper dermatitis and perineal injuries occurred frequently in NICUs from North America and in NICUs using petrolatum-based ointment. Perineal injuries (injury of any nature specific to the perineal region) were less in NICUs that assessed skin at least every four hours, had a local skincare guideline or used the Braden Q tool. Although Braden Q tool is widely used for pressure injury risk assessment, its association with lower odds of perineal injuries in our survey could be explained by pressure injury at the perineum [4].

Medical devices can cause pressure injuries [20]. The pressure injury sites reported in this survey are consistent with previous reports [5, 21]. Preventing pressure injury and pressure ulcer is essential, as they affect the patient and the organization [22]. The evidence for pressure injury prevention strategies in EP infants is limited [23–25]. Frequent surveillance, rotating the site of medical devices, routinely changing body position, use of pressure injury prevention devices or special mattresses, alcohol-free products and petrolatum-based ointments are strategies to prevent pressure injuries in newborn infants at high risk of skin injuries [4]. But these practices are often extrapolated from adult and/or pediatric literature [26]. Regular skin assessment, at least every 12 hours, is suggested for the early identification of pressure injuries from medical devices [24, 27]. In this study, diaper dermatitis, pressure, perineal and abrasion injuries were less when skin assessments were performed at least every four hours.

Using topical skin cleansing agents prior to invasive procedures reduces hospital-acquired bloodstream infections [28]. While most NICUs used a topical cleansing agent, the choice of cleansing agent varied, and few used sterile water. In adults, the application of a topical chlorhexidine-based agent is possibly superior to povidone-iodine in reducing catheter-related bloodstream infections [29]. But evidence for its superiority over other agents in EP infants is lacking [30, 31]. Hence, the Centers for Disease Control and Prevention makes no such recommendation for its use in infants < 2 months of age. There are safety concerns regarding systemic absorption of iodine and alcohol-based cleansing solutions and lack of information on long-term neurodevelopment especially as infants born at 22 weeks GA are offered active care [28, 32–35]. Maintaining skin integrity and reducing catheter-related bloodstream infections is vital for their survival. Hence, the question of which cleansing agent is superior in efficacy and safety for EP infants should be addressed.

While daily or more frequent skin assessments are suggested, there is ambiguity regarding its optimal frequency, and its effect on occurrence of skin injury [4]. Most NICUs were performing them at least every four hours and this practice was associated with less skin injuries. It is important to use a valid skin assessment tool to assess skin health objectively. Most skin assessment tools used either did not account for prematurity or were not validated for use in preterm infants [36]. Newer tools for evaluating skin integrity are reported [37, 38]. However, further testing of these tools in EP infants is suggested before making changes in practice [4]. Interestingly, we observed that abrasion injuries were less when NICUs used even a local skin assessment tool compared to none. This highlights the need for use of an objective skin surveillance tool. In this survey, 28% of NICUs did not have a local skincare guideline. Integrating a skincare guideline into practice probably reduces skin injuries by delivering evidence-based care, improving staff education, and reducing variations in practice.

Application of emollients may benefit term infants, but debate continues regarding the benefits for preterm infants [39]. In this survey, oil-based emollient was used most often and a quarter of NICUs reported interference with medical device adherence as the most common complication. Income status-based and region-based variation was observed for application of emollient and complications from its use. Additionally, NICUs applying petroleum-based emollient reported a higher odds of skin injuries. The true reason for this observation needs further exploration. Plausible reasons could include skin barrier disruption by the process of emollient application (e.g., massaging), increased risk of skin colonization and infection from pathogens, and adverse local and systemic effects form absorption of chemicals contained in the emollient [39]. Skin protection from the application of coconut oil has been reported, but concerns have been raised regarding interference with medical device adherence and systemic infection [40–42]. Applying emollients (e.g., sunflower or coconut oil) to preterm infants in LMICs improved weight gain and reduced sepsis [43, 44]. A randomized trial is currently investigating the effect of topical coconut oil application on the development of sepsis in EP infants [45].

Umbilical cord care practices reported by most NICUs aligned with the current international recommendation [46]. Geographic and resource-settings-based variation in using a topical drying agent was observed. Keeping a wound clean and dry, using sterile water for wound cleaning, and applying hydrocolloid dressings were the most consistent practices that aligned with wound management principles [4, 47]. Evidence to support routine application of topical antimicrobial agents for wound healing is lacking. Antiseptic skin cleansers were used by 31% of NICUs. This practice can cause trauma to the healing tissue and delay wound healing [47, 48]. Application of silicone-based or hydrocolloid-based adhesive dressings promotes wound healing and reduces trauma caused by removal of the adhesive [11, 49]. Despite a lack of similar evidence in EP infants, hydrocolloid-based and silicone-based dressings are used. There is emerging evidence of the safety and efficacy of *Leptospermum* honey in preterm infants, but this needs further exploration in controlled trials [50, 51].

Previous studies have focused on practices within a country [52, 53]. The strength of this study was representative participation from all geographic regions and resource settings, therefore the findings are generalizable to a wider neonatal community. Our study has certain limitations. The questionnaire was prepared only in the English language; this may have excluded participation of NICUs from non-English speaking regions. The COVID-19 pandemic may have affected participation in the survey. Although participating unit’s identifiable information was not recorded, we are confident that duplicate responses from the same unit were not included by checking the demographic data and the survey responses. Finally, an overall survey response rate was not reported, as the total number of NICUs from each participating region was not known.

In conclusion, skin injuries were common in EP infants. Having a local skincare guideline and performing skin assessments at least every four hours were associated with reduced odds of skin injuries. Further evidence on skincare practices in EP infants is needed to formulate region and resource settings-based guidelines, which will reduce variations in practices. Future research may inform strategies on reducing skin injuries and delivering a better quality of health care, leading to improved clinical outcomes.

## Data Availability

All data on skincare practices generated or analyzed during this study are included in this published article.
